# Supplementary material for: Analysis of the Potassium-Solubilizing Priestia megaterium Strain NK851 and Its Potassium Feldspar-Binding Proteins
Source: Int J Mol Sci. 2023 Sep 18;24(18):14226. doi: 10.3390/ijms241814226 (PMC10531590; doi:10.3390/ijms241814226)
Supplement: Supplementary file 1 [file ijms-24-14226-s001.zip › ijms-2450534-supplementary.pdf]

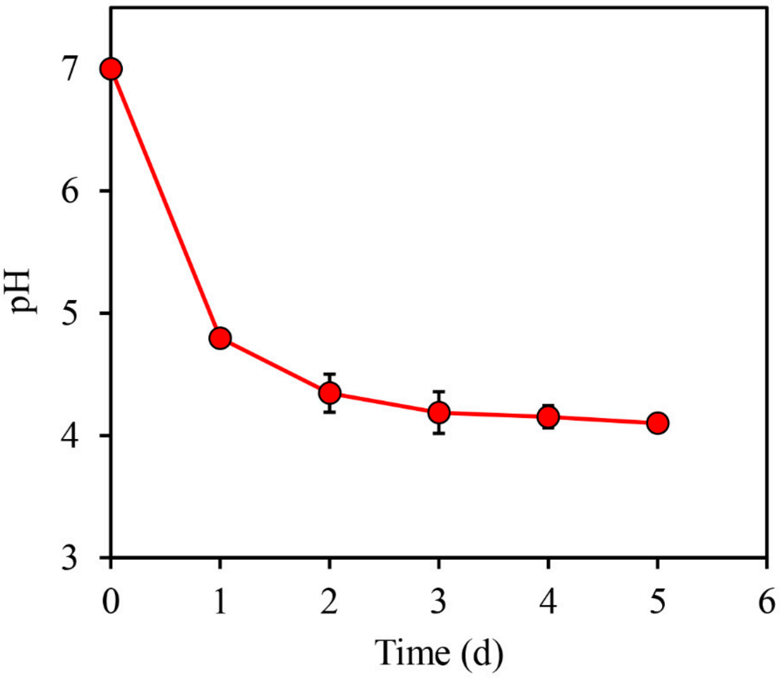

Figure S1. Change of the pH values of the NK851 cultures in different days. The strain was cultured in potassium-solubilizing medium for indicated time, followed by pH measurement.
